# Fear of childbirth in a sample of Tunisian women: factors related to pregnancy

**DOI:** 10.1192/j.eurpsy.2024.1675

**Published:** 2024-08-27

**Authors:** M. Abdelkefi, R. Feki, R. Walha, W. Zid, I. Gassara, N. Smaoui, S. Omri, N. Charfi, L. Zouari, J. Ben thabet, K. Chaabene, M. Maalej bouali, M. Maalej

**Affiliations:** ^1^Psychiatry C department; ^2^Gynecology-Obstetrics department, Hedi Chaker university hospital, sfax, Tunisia

## Abstract

**Introduction:**

Fear of childbirth is attracting growing interest because of its impact on the experience of pregnancy and on the progress of childbirth and it seems that some women are more susceptible to fear of childbirth than others are.

**Objectives:**

Our objective is to identify pregnancy factors that predict the fear of childbirth.

**Methods:**

We approached 350 pregnant women consulting at the Gynecology-Obstetrics department of the Hedi Chaker University Hospital of Sfax. We collected their sociodemographic and clinical data. Fear of childbirth was assessed using the French version of the Traumatic Event Scale (TES), adapted to assess fear of childbirth.

**Results:**

The mean age of the participants was 28 years (16-41) and the mean gestational weak was 36.27. Half of the participants (53.7%) were nulliparous, and eight reported a history of infertility. The pregnancy was not planned in 61% of cases. As many as 67% of the participants had regular checkups, 50.3% had exaggerated somatic symptoms and 34.3% had pregnancy-related diseases.

The mean score for the TES was 48.73 ± 13.72.

We found a positive correlation between the TES score and nulliparity (p=0.01), gestational age ≥ 40 weeks (p=0.01), planned pregnancy (p=0.002), exaggerated somatic symptoms (p=0.03), and pregnancy-related diseases (p<0.001).

**Conclusions:**

Identification of women at risk for fear of childbirth could help in preparing them before or during pregnancy to improve their childbirth experiences.

**Disclosure of Interest:**

None Declared

